# Modulation of mdm2 pre-mRNA splicing by 9-aminoacridine-PNA (peptide nucleic acid) conjugates targeting intron-exon junctions

**DOI:** 10.1186/1471-2407-10-342

**Published:** 2010-06-30

**Authors:** Takehiko Shiraishi, Jonhard Eysturskarð, Peter E Nielsen

**Affiliations:** 1 Department of Cellular and Molecular Medicine, The Panum Institute, University of Copenhagen, Health Science Faculty, Blegdamsvej 3c, 2200 Copenhagen N, Denmark

## Abstract

**Background:**

Modulation of pre-mRNA splicing by antisense molecules is a promising mechanism of action for gene therapeutic drugs. In this study, we have examined the potential of peptide nucleic acid (PNA) 9-aminoacridine conjugates to modulate the pre-mRNA splicing of the mdm2 human cancer gene in JAR cells.

**Methods:**

We screened 10 different 15 mer PNAs targeting intron2 at both the 5' - and the 3'-splice site for their effects on the splicing of mdm2 using RT-PCR analysis. We also tested a PNA (2512) targeting the 3'-splice site of intron3 with a complementarity of 4 bases to intron3 and 11 bases to exon4 for its splicing modulation effect. This PNA2512 was further tested for the effects on the mdm2 protein level as well as for inhibition of cell growth in combination with the DNA damaging agent camptothecin (CPT).

**Results:**

We show that several of these PNAs effectively inhibit the splicing thereby producing a larger mRNA still containing intron2, while skipping of exon3 was not observed by any of these PNAs. The most effective PNA (PNA2406) targeting the 3'-splice site of intron2 had a complementarity of 4 bases to intron2 and 11 bases to exon3. PNA (2512) targeting the 3'-splice site of intron3 induced both splicing inhibition (intron3 skipping) and skipping of exon4. Furthermore, treatment of JAR cells with this PNA resulted in a reduction in the level of MDM2 protein and a concomitant increase in the level of tumor suppressor p53. In addition, a combination of this PNA with CPT inhibited cell growth more than CPT alone.

**Conclusion:**

We have identified several PNAs targeting the 5'- or 3'-splice sites in intron2 or the 3'-splice site of intron3 of mdm2 pre-mRNA which can inhibit splicing. Antisense targeting of splice junctions of mdm2 pre-mRNA may be a powerful method to evaluate the cellular function of MDM2 splice variants as well as a promising approach for discovery of mdm2 targeted anticancer drugs.

## Background

Antisense molecules with significantly modified backbones such as peptide nucleic acids (PNA), methoxyethoxy (MOE) and locked nucleic acids (LNA), or morpholino oligos, rendering them RNase H inactive, are able to modulate mRNA splicing when targeting intron-exon junctions in pre-mRNA [1-7]. For instance correction of aberrant splicing by blocking cryptic 5'- or 3'- splice sites, induction of exon skipping [8-10] and force selection of an alternative splice site by targeting antisense molecules to original splice sites [11] have been demonstrated. Thus antisense targeting of splice junctions have the potential of inducing shifts in the ratio between biologically functional splice variants or even induce non-natural splice variants with novel biological function of the resulting protein. Therefore, splicing targeting technology may open a range of opportunities for gene targeting in drug discovery and molecular biology contexts [5,12,13].

The mdm2 oncogene is amplified and/or over expressed in several cancer types [14]. This oncogene encodes a protein that negatively controls the functions of the p53 tumor suppressor protein by blocking the transactivation domain and by stimulating the degradation of p53. Down regulation of MDM2 has been recognized as a potential mechanism for cancer therapy [15,16] because down-regulation of MDM2 in tumors exhibiting MDM2 over-expression should induce p53 stability and thus sensitization to DNA-damaging treatments via p53-dependent pathways [17-20]. Accordingly, recent studies have shown down regulation of full-length MDM2 protein through a traditional RnaseH dependent antisense approach. In addition, more than 40 different splice variants of mdm2 mRNA have been detected in tumors and normal cells [17,21], but the potential functions or oncogenic properties of the different MDM2 isoforms are far from fully understood [22-24]. Therefore, targeting of mdm2 mRNA splicing could be an effective way of controlling and studying overall MDM2 expression and function.

It has recently been shown that PNA oligomers targeted to exon-intron splice junctions are potent inhibitors/modulators of mRNA splicing [6,25]. By targeting a 3'- splice site, at least two outcomes have been found although no systematic studies have yet been published. The spliceosome will either skip the exon and thus produce a truncated mRNA missing the exon, or skip the intron excision and thereby produce a larger mRNA still containing the intron. Therefore, PNA molecules designed to down-regulate full length mdm2 mRNA or shift relative populations of splice variants by splicing modulation may be a useful approach for both future therapy as recently indicated for PNA targeting of CD40 pre-mRNA (7), as well as for investigating the functions of mdm2 splice variants [17]. However, limited information is available concerning the optimum design of antisense PNA oligomers, including target location, for the desired splicing modulation [6,7,26]. Therefore we decided to perform a more systematic study of PNA oligomers targeting the 5'- or 3'-splice sites in the human MDM2 gene.

In this study, we have tested ten PNAs (15-mer) targeting intron2 (seven PNAs targeting the 3'-splice site and three PNAs are targeting to the 5'-splice site) as well as a PNAs targeting the 3'-splice site of intron3. Depending on the target site intron and/or exon skipping was observed, and most interestingly targeting of intron2 also affected the splicing of intron3. Finally, treatment of JAR cells with the PNA targeting intron3 resulted in a significant reduction in the level of MDM2 protein and a concomitant increase in the level of tumor suppressor p53

## Methods

### Synthesis of PNA

The sequences of the PNAs and nomenclatures are listed in Table 1. PNA synthesis was carried out as reported in elsewhere [27]. PNAs were HPLC-purified and characterized by mass spectrometry. N-(9-aminoaciridinyl)-6-aminohexanoic acid (Acr) was covalently linked to the PNA at the N-terminal of the PNA [20] through an ethyleneglycol type linker (eg1, 8-amino-3,6-dioxaoctanoic acid) (Acr-PNA). Purified PNAs were lyophilized and stored at 4°C until use.

**Table 1:** Nomenclatures and sequences of PNAs

Target ^(a)^	PNA No.	Sequence ^(a)^
5'-Int2	PNA2545	H-Acr-eg1- TCG GTG CTT ACC TGG-NH_2_
	PNA2547	H-Acr-eg1-TGC TTA CCT GGA TCA-NH_2_
	PNA2549	H-Acr-eg1-TTA CCT GGA TCA GCA-NH_2_
		
3'-Int2	PNA2551	H-Acr-eg1-TGT TGG TAT TGC ACA-NH_2_
	PNA2553	H-Acr-eg1-TGG TAT TGC ACA TTT-NH_2_
	PNA2555	H-Acr-eg1-TAT TGC ACA TTT GCC-NH_2_
	PNA2406	H-Acr-eg1-TGC ACA TTT GCC TAC-NH_2_
	PNA2557	H-Acr-eg1-ACA TTT GCC TAC AAG-NH_2_
	PNA2559	H-Acr-eg1-TTT GCC TAC AAG GAA-NH_2_
	PNA2561	H-Acr-eg1-GCC TAC AAG GAA AAA-NH_2_
		
3'-Int3	PNA2512	H-Acr-eg1-TTT GGT CTA ACC TAT -NH_2_
	PNA2967	H-(D-Arg)_8_-TTT GGT CTA ACC TAT-NH_2_
	PNA2968	H-(D-Arg)_8_-Lys(Deca)-TTT GGT CTA ACC TAT-NH_2_
		
PNA2406 mismatch	PNA2681	H-Acr-eg1-TGC A**G**A TTT **C**CC TAC-NH_2_
PNA2512 mismatch	PNA2733	H-Acr-eg1-TTT **A**GT CTA **G**CC TAT -NH_2_

^(a) ^Three PNAs are targeted to the 5'- splice site of intron2 of mdm2 pre-mRNA (5'-Int2). Seven PNAs are targeted to the 3'-splice site of intron2 (3'-Int2). PNA 2512 is targeted to the 3'- slice site of intron3 (3'-Int3). PNA2681 and PNA2733 are mismatch control PNAs for PNA2406 and PNA2512, respectively. Mismatches are indicated in bold. ^(b) ^The sequences of the PNAs (15-mer) are written from N-terminal to C-terminal-end. Acr and eg1 indicate 9-aminoacridinyl ligand and ethyleneglycol linker (8-amino-3,6-dioxaoctanoic acid), respectively.

### Cell culture

JAR cells were provided by Dr. Peter Ebbesen (The Stem Cell Laboratory, Aalborg University, Denmark). The cells were cultured in RPMI-1640 medium (Sigma) supplemented with 10% fetal bovine serum (FBS), 1.5% glutamax (Gibco) and streptomycin/penicillin (100 U/ml each)) at 37°C in a humidified atmosphere of 95% air and 5% CO_2_.

### PNA transfection

**Figure 1:**
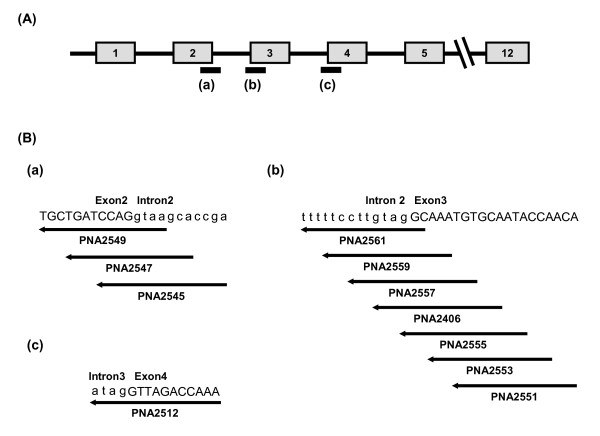
**The MDM2 gene and PNA target positions**. (A) The regions of mdm2 pre-mRNA used as targets for PNAs are indicated with thick bars under the exons ((a), (b) and (c)). Numbered boxes: exons; lines: introns. (B) The exact sequence position of PNAs targeted to the 5'-splice site of intron2 (a), 3'-splice site of intron2 (b) and 3'-splice site of intron3 (c) are shown. Intron sequences are in lower case.

Exponentially growing cells were cultured in 6 well plates the day before transfection to give 40-60% confluency at the time of transfection. LipofectAMINE (LFA, Gibco) or LipofectAMINE2000 (LFA2000, Gibco) was used for the transfection of the PNAs with Acr modification. LFA was used unless otherwise stated. The PNA solution (200 μM in water) was heated for 2 minutes at 90°C then cooled to room temperature and used as stock solution. In a separate tube, LFA or LFA2000 was diluted with OPTI-MEM (Gibco) at a ratio of 120 μl/ml and 80 μl/ml, respectively. Then the diluted LFA or LFA2000 solution was mixed with an equal volume of the PNA solution and incubated for 5 min, and finally 200 μl Acr-PNA/LFA solution was mixed with 800 μl of OPTI-MEM or the RPMI medium (10% FBS, 1% glutamax) respectively for LFA and LFA2000. This PNA transfection solution was added to the cells after removing the growth medium using 0.5 ml or 0.1 ml/well for in 24 well plate or 96 well plate, respectively. After 3-6 h incubation, the cells were supplemented with 1 ml of growth medium (RPMI1640 containing 10% FBS and 1.5% glutamax) and incubated further up to 24 h. The PNA2967 and PNA2968 (PNAs with oligoarginine) were transfected without cationic lipids and 120 μM chloroquine (CQ) was added to the OPTI-MEM for the CQ treatment. Nomenclatures and sequences of PNAs are listed in table 1. The positions of the PNA target sites are illustrated in Figure 1.

### RT-PCR analysis

24 h after transfection, total RNA was extracted from the cells by using RNeasy Mini kit (Qiagen) and subjected to RT-PCR. RT-PCR was performed using the OneStep RT-PCR kit (Qiagen). 4 ng of total RNA was used for each RT-PCR reaction (20 μl). Primers for RT-PCR detection of splice sites of mdm2 pre-mRNA and β-actin (as internal control) are listed in Table 2. The RT-PCR program was as follows: (55°C, 35 min) × 1 cycle, (95°C, 15 min) × 1 cycle, (94°C, 1 min; 55°C, 1 min; 72°C, 1 min) × 26-30 cycles. RT-PCR products were analyzed on 2% agarose gels with 1 × TBE buffer and visualized by ethidium bromide staining. Gel images were captured by ImageMaster (Pharmacia Biotech) and analyzed by UN-SCAN-IT software (Silk Scientific Corporation). It is important to note that the RT-PCR experiments cannot be used for absolute quantification from one set of experiments to the next due to variations in RNA extraction, RT-PCR amplification and gel analysis. Thus the results are reported relative to the internal control (β-actin).

**Table 2:** Nomenclatures of primer sets and sequences of RT-PCR primers.

Primer set^(a),^	Primer name	sequence (5'→3')	Product size (bp)	Remarks
A	Exo2S	CGATTGGAGGGTAGACCTGT	161	intron2 5'-splice site
	Int2AS	CACGATGAAAACTGGAAATCA		
				
B	Int2S^a^	TGCTTGTAGCTTTAGTTTTA	90	intron2
	Int2AS	CACGATGAAAACTGGAAATCA		
				
C	Int2S^b^	GATTTCCAGTTTTCATCGTGT	120	intron2 3'-splice site
	Exo3AS	GGTCTCTTGTTCCGAAGCTG		
				
D	Exo3S	CAGCTTCGGAACAAGAGACC	169	intron3 5'-splice site
	Int3AS	GCAGTTACGCCAGAGGTAGC		
				
E	Int3S	TCTTTGCTCTTTTGGATTGGA	251	intron3 3'-splice site
	Exo4AS	TTTTTGTGCACCAACAGACTTT		
				
F	Exo4S	AAGCCATTGCTTTTGAAGTTATT	151	intron4 5'-splice site
	Int4AS	GCCAATTTCTCCACATGGTC		
				
G	Int4S	TGGTTCCTGGTTGTTTACCC	215	intron5 3'-splice site
	Exo5AS	CACGCCAAACAAATCTCCTA		
				
H	ActF1	CCCTGGAGAAGAGCTACGAG	223	β-actin (771-993)
	ActR1	ATGCCAGGGTACATGGTGGT		
				
I	ActF2	CTTCCTGGGCATGGAGTC	166	β-actin (862-1027)
	ActR2	CAGGGCAGTGATCTCCTTCT		

^(a)^A-H indicates the target sequences for RT-PCR within the mdm2 pre-mRNA. For primer set H and I, location of the amplification products within the β-actin (Accsession#, BC001301) are indicated.

### Western blot analysis

Cells were transfected with PNA as described above and subjected to protein sample preparation: The cells were lysed in boiling lysis buffer (1% SDS, 10 mM Tris-HCl, pH 7.2, 1X protease inhibitor cocktail tablet (Roche, Germany)), and 40 μg of the protein was fractionated by SDS-polyacrylamide gel electrophoresis (SDS-10% PAGE) and transferred to a PVDF-membrane (ADVANTEC MFS). Blocking, detection and re-probing were performed with the ECL-Plus system (Amersham) following the manufacture's instruction. Briefly, the PVDF-membrane was blocked with 5% skim milk (in 20 mM Tris-HCl, pH 7.2, 150 mM NaCl, 0.1% Tween 20) over night at 4°C and incubated with a primary antibody (anti-MDM2, SMP14 (Sigma); anti-p53, ab6 (Santa Cruz); anti-β-actin, AC-15, (Sigma)) overnight at 4°C. The membrane was washed with washing buffer (20 mM Tris-HCl, pH 7.2, 150 mM NaCl, 0.01% Tween 20) three times for a minimum of 15 min each at room temperature, and then incubated with 1:20000 diluted anti-mouse IgG-horse radish peroxidase conjugated secondary antibody (DAKO, P0161) for 1 h at room temperature. After washing as described above, the protein of interest was detected with ECL-Plus (Amersham).

### Cytotoxicity test

JAR cells, plated in a 96 well plate the day before transfection, were subjected to PNA transfection in combination with camptothecin (CPT) treatment. CPT was added to the PNA transfection solution at the desired concentration and incubated for 48 h. Subsequently, cell viability was determined by the MTS-assay using the CellTiter 96 Aqueous Non-Radioactive Cell Proliferation Assay (Promega) according to the manufacturer's instructions. The absorbance is presented as relative cellular viability (absorbance from non-PNA treated cells was set as 100%).

## Results and Discussion

Ten PNAs targeting intron2 at the 5' or 3' splice sites were tested for their ability to inhibit splicing of mdm2 pre-mRNA. As the first in frame AUG translation start codon is located in exon3, we focused on splice interference that might result in exon3 skipping, thereby prohibiting any translation initiation from the mRNA. In order to facilitate cellular uptake, we used 9-aminoacridine conjugated PNAs in combination with cationic lipid transfection reagent as previously described [20].

**Figure 2:**
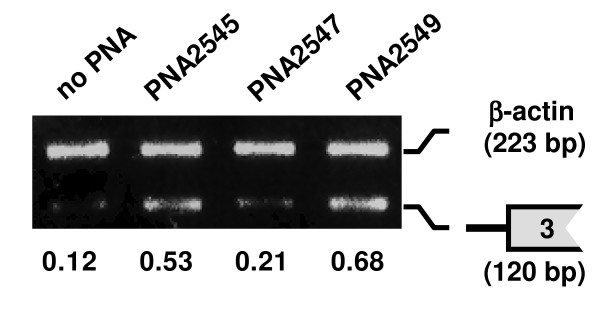
**Effect of PNAs on the splicing of intron2 in mdm2 pre-mRNA**. Splicing inhibition of three PNAs (PNA2545, 2547 and 2549) targeted to the 5'-splice site of intron2 in mdm2 pre-mRNA. JAR cells were transfected with PNA (2 μM) for 24 h and subjected to RT-PCR analysis by using extracted total RNA. RT-PCR was performed with primer sets C and H (see Table 2), for 3'-splice site of the intron2 (120 bp) and β-actin (223 bp) (as internal control), respectively. Results of RT-PCR with 27 cycles are shown. The numbers under the figure indicate the relative amount (normalized to β-actin) of the target mdm2 splicing variant.

Three PNAs (PNA 2545, 2547 and 2549), targeting the 5'-splice site of intron2 (Figure 1(a)), were tested at 2 μM. Splicing inhibitory effects were analyzed by RT-PCR amplifying a region covering the 3'-splice site of intron2 using primer set C (primer sets are listed in Table 2) to detect mRNA still including intron2. The 3'-splice site was used instead of the 5'-splice site to avoid RT-PCR inhibition by PNA targeting the 5'-splice site. PNA2549 (complementary to 11 bases of exon2 and 4 bases of intron2) showed the highest splicing inhibition among the three nested PNAs, and PNA 2547 did not show any significant splicing inhibition (Figure 2). Exon skipping was not detected for any of these PNAs (at 2 μM concentration).

**Figure 3:**
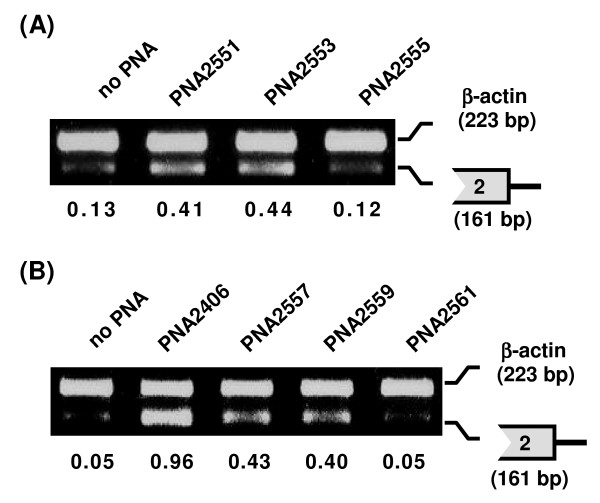
**Effect of PNAs on the splicing of intron2**. Splicing inhibition of intron2 in mdm2 pre-mRNA by seven PNAs (PNA2551, 2553, 2555, 2406, 2557, 2559 and 2561) targeted to the 3'-splice site of intron2. JAR cells were transfected with PNA (2 μM) for 24 h and subjected to RT-PCR analysis by using extracted total RNA. RT-PCR was performed with primer sets A and H (see Table 2), for the 5'-splice site of the intron2 (161 bp) and β-actin (223 bp) (as internal control), respectively. RT-PCR results with 27 PCR cycles for three PNAs (PNA2551, 2553 and 2555) (A) and four PNAs (PNA2406, 2557, 2559 and 2561) (B) are shown. The numbers under the figure indicate the relative amount (normalized to β-actin) of the target mdm2 splicing variant.

**Figure 4:**
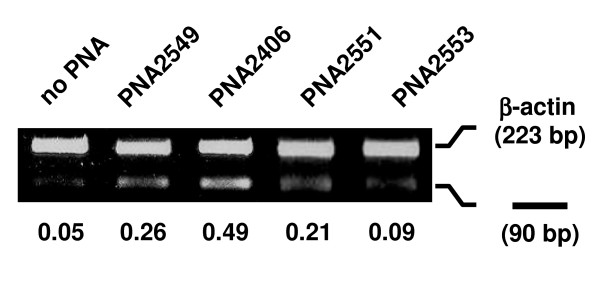
**Effect of PNAs on the splicing of intron2**. Comparison of PNAs 2549, 2406, 2551 and 2553 in terms of splicing inhibition of intron2 in mdm2 pre-mRNA. JAR cells were transfected with PNAs (2 μM) for 24 h and subjected to RT-PCR analysis using extracted total RNA. RT-PCR was performed with primer sets B and H (see Table 2), for intron2 in mdm2 pre-mRNA (90 bp) and β-actin (223 bp) (as internal control), respectively. RT-PCR results with 27 PCR cycles are shown. The numbers under the figure indicate the relative amount (normalized to β-actin) of the target mdm2 splicing variant.

The effects of seven PNAs targeting the 3'-splice site of intron2 (Figure 1(b)), were compared by RT-PCR amplification of a region including the 5'-splice site of intron2 (Figure 3). The PNAs were tested at 2 μM concentration and of the seven PNAs, one PNA (2406) was clearly more active, four PNAs (2551, 2553, 2557 and 2559) did inhibit splicing to a significant degree, while two PNAs (2555 and 2561) showed no activity (same as no PNA control). A direct comparison between PNAs 2406, 2549, 2551 and 2553 clearly showed the highest activity for PNA 2406 (Figure 4), and we therefore decided to focus further studies on this PNA. The limited data also indicate that the most active PNAs for a given splice junction (PNA 2549 and 2406) should be positioned over the junction with a bias (75%) to the exon side.

**Figure 5:**
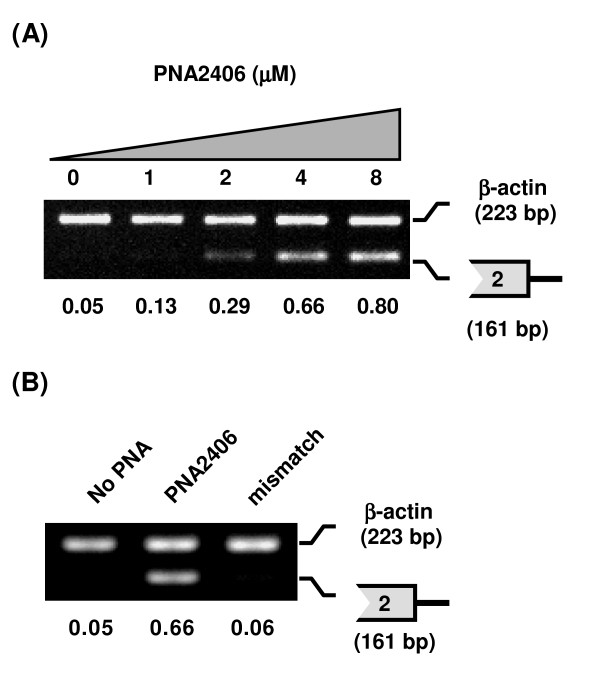
**Effect of PNA2406 on the splicing of intron2**. JAR cells were transfected with PNAs at the indicated concentrations for 24 h and subjected to RT-PCR analysis using extracted total RNA. RT-PCR was performed with primer sets A and H (see Table 2) for 5'-splice site of intron2 in mdm2 pre-mRNA (161 bp) and β-actin (223 bp) (as internal control), respectively. The numbers under the figure indicate the relative amount (normalized to β-actin) of the target mdm2 splicing variant. A, Dose dependent splicing inhibition of intron2 by PNA2406 (1, 2, 4 and 8 μM). 26 PCR cycles was used. B, comparison of PNA2406 with its mismatch PNA (PNA2681, mismatch) holding two mismatches (at 2 μM). 27 PCR cycles was used.

**Figure 6:**
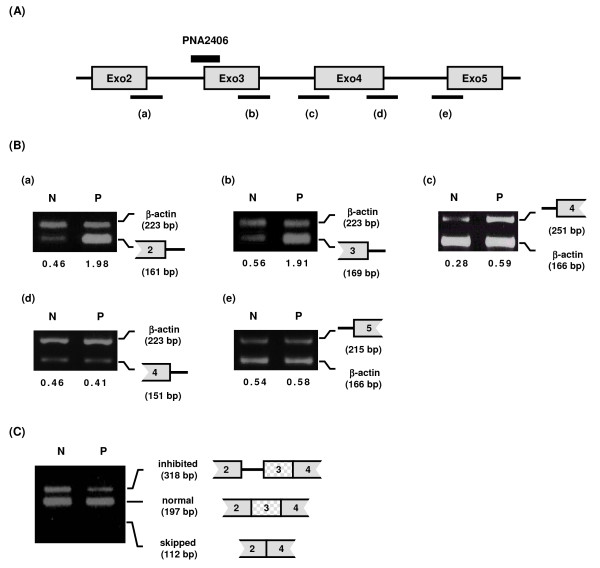
**Effect of PNA2406 on the splicing of introns 2, 3 and 4**. JAR cells were transfected with PNA2406 at 2 μM for 24 h and subjected to RT-PCR analysis using the extracted total RNAs. N: no PNA treatment. P: treated with PNA2406 (2 μM). (A) Location of the PNA2406 and splice sites analyzed ((a), (b), (c), (d) and (e)) are indicated. (B) The structures or names of RT-PCR products are shown on the right of each picture. Primer sets for the detection of each splice sites (in Figure 6A) by RT-PCR (product length) are as follows: (a), A (161 bp) and H (β-actin, 223 bp); (b), D (169 bp) and H (β-actin, 223 bp); (c), E (251 bp) and I (β-actin, 166 bp); (d), F (151 bp) and H (β-actin, 223 bp); (e), G (215 bp) and I (β-actin, 166 bp). The numbers under the figure indicate the relative amount (normalized to β-actin) of the target mdm2 splicing variants. (C). Primers, Exo2S and Exo4AS in Table 2, were used for RT-PCR for the detection of exon3-skipped form. Locations of the splice inhibited form (inhibited (318 bp)), normal splice form (normal (197 bp)) and exon3-skipped form (skipped (112 bp)) although the last one (skipped form) was not visible.

Inhibition of intron2-exon3 cleavage by PNA2406 exhibited a clear dose response (Figure 5A), and a control PNA (2681) having two mismatches in the sequence (by nucleobase interchange) in contrast to PNA 2406 showed no activity at 2 μM (Figure 5B). Next we asked whether blocking of the intron2-exon3 junction by PNA 2406 had any other effects on splicing such as skipping of exon3 or inhibition of the splicing of adjacent introns. These experiments were performed by RT-PCR using the primers indicated in Figure 6A. The results (Figure 6C) very clearly show no indication of significant skipping of exon3 (112 bp). Because this sequence does not include the PNA target, the RT-PCR reaction is not inhibited by the PNA. In contrast the splicing inhibited product (318 bp) does contain the PNA target, and this is decreased in the PNA treated sample. Also the effect of the PNA is note solely localized to the targeted intron but does affect splicing of adjacent introns. For example the amount of unspliced intron 3 is increased 2-4-fold (Fig 6(B) (b) and 6(c)), while no significant change of intron 4 splicing was detected (Figure 6 (B), (d) and 6(e)).

**Figure 7:**
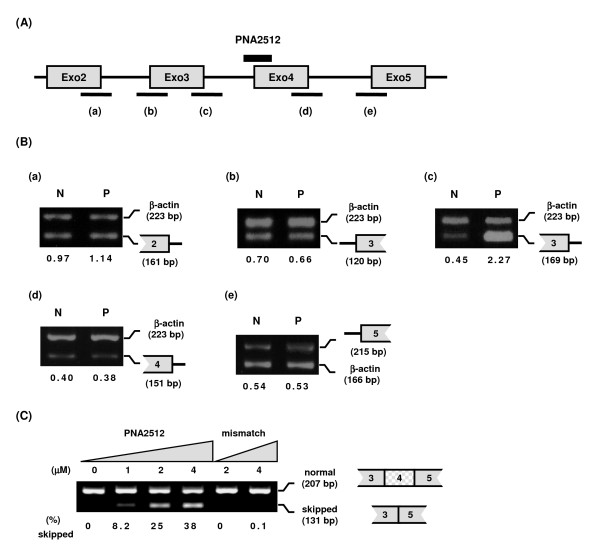
**Effect of PNA2512 on the splicing of introns 2, 3 and 4**. JAR cells were transfected with PNA2512 or mismatch PNA (PNA2733) for 24 h and subjected to RT-PCR analysis using extracted total RNA. (A) Location of PNA2512 and detected splice sites ((a), (b), (c), (d) and (e)) are indicated. (B) Cells were transfected with PNA2512 (2 μM) and subjected to RT-PCR analysis. N: no PNA treatment. P: treated with PNA2512 (2 μM). The structures or names of RT-PCR products are shown on the right of each picture. Primer sets (see table 2) for the detection of each splice site by RT-PCR (product length) are as follows: (a), A (161 bp) and H (β-actin, 223 bp); (b), C (120 bp) and H (β-actin, 223 bp); (c), D (169 bp) and H (β-actin, 223 bp); (d), F (151 bp) and H (β-actin, 223 bp); (e), G (215 bp) and I (β-actin, 166 bp). The numbers under the figure indicate the relative amount (normalized to β-actin) of the target mdm2 splicing variants. (C) Effect of PNA2512 on the skipping of exon4. Cells were treated with PNA2512 or its mismatch PNA (PNA2733) at the indicated concentrations and subjected to RT-PCR analysis. For the RT-PCR, Exo3S and Exo5AS (primers in Table 2) were used. Locations of the normally spliced form (Normal (207 bp)) and exon4-skipped form (skipped (131 bp)) are indicated on the right. Numbers under each lane indicate the amount of the skipped form relative to the sum of skipped and normal form.

**Figure 8:**
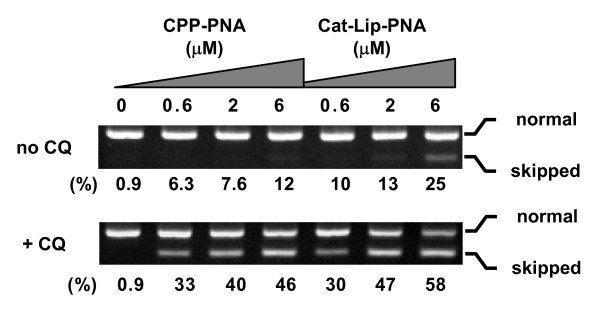
**Effect of CPP-PNA (PNA2967) and Cat-Lip-PNA (PNA2968) on the skipping of exon4**. JAR cells were incubated with PNA for 4 h in the serum free OPTI-MEM medium (without cationic lipids) in the absence or the presence of 120 μM chloroquine (CQ) and incubated further for 24 h after supplemented a growth medium to have 10% FBS concentration. Total RNA was extracted and subjected to RT-PCR using Exo3S and Exo5AS primers (see Table 2). Locations of the normally spliced form (Normal, 207 bp) and exon4-skipped form (Skipped, 131 bp) are indicated on the right of the pictures. Numbers under each lane indicate the amount of the skipped form relative to the sum of skipped and normal form.

To validate the target design of PNA for efficient splicing inhibition, we designed another PNA (PNA2512) targeting the 3'-splice site in intron3 with a complementary of 4 bases in intron3 and 11 bases in exon4 (Figure 1C). The effect of PNA2512 on splicing at intron2, 3 and 4 was analyzed by detecting the regions including the 5' or 3' splice sites of intron2, 3, and 4 (Figure 7A, region (a) - (e)). Only inhibition of splicing out of intron3 was observed in this case. Furthermore, in contrast to PNA2406, PNA2512 induced skipping of exon4 in addition to intron skipping (Figure 7B-(c), Figure 7C). PNA2512 showed a dose-dependent increase of the skipped form and decrease of the normal form (ca 1/3 conversion at 4 μM), while there was no such effect with the mismatch PNA. This PNA oligomer was further studied by using a different cellular delivery method not requiring cationic lipid assistance, but based on cell penetrating peptides (CPP). In this case the PNA was conjugated to octaarginine (PNA2967) or to decanoyl-octaarginine, having decanoic acid (as a lipidic domain) attached to the ε-amine of Lys side chain (Cat-Lip-PNA [28], PNA2968). These PNAs were transfected to the cells both in the absence or the presence of the endosomolytic reagent chloroquine (CQ) since CQ is known to improve the cellular activity of CPP conjugates (Figure 8) [29]. The PNAs showed exon skipping activity in a dose dependent manner and the effect was significantly improvement upon CQ addition. As expected (28) the Cat-Lip PNA2968 exhibited the higher activity reaching more than 50% conversion at 6 μM in the presence of CQ, Although the activity of this PNA in the absence of CQ - somewhat surprisingly - is lower than that exhibited by lipofectamine mediated delivery of PNA2512, it is still higher than that observed for the analogous arginine PNA2967. Furthermore, CPP-PNA mediate splicing modulation is of considerable interest, since CPP delivery in contrast to lipofectamine mediated delivery holds some promise for *in vivo *and eventually therapeutic use [30,31].

**Figure 9:**
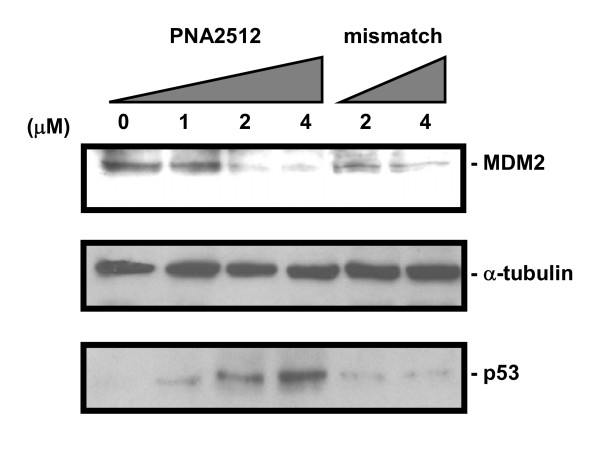
**Effect of PNA2512 on the protein level of MDM2 and p53**. JAR cells were transfected with PNA 2512 or its mismatch PNA (PNA2733) and subjected to western blot analysis. Equal amount of protein were separated on SDS-PAGE and transferred to PVDF membrane. The membrane was probed with monoclonal antibody for MDM2 and reacting proteins were detected using the ECL-Plus system (Amersham). The membrane was washed and re-used for detection with a monoclonal antibody of p53 or α-tubulin. The numbers under the figure indicate the relative amount (normalized to α-tubulin) of the target proteins.

Because PNA 2512 produced a combination of intron and exon skipped mRNA forms both of which would prohibit correct protein translation, we decided to analyze the effect of treatment with this PNA on the protein expression level of mdm2 as well as of p53, which is directly regulated by mdm2. After PNA treatment, cellular proteins were subjected to western blot analysis. As shown in Figure 9, PNA2512 induced a significant, dose dependent decrease of MDM2 (90 kDa) protein levels at 2 μM and 4 μM (while mismatch PNA (PNA2733, with two base pairs internal exchange) clearly induced less decrease of the MDM2 than full matched PNA at the same concentrations). No evidence for accumulation of any truncated MDM2 protein from the truncated mRNA missing exon4 (mw 52374) or from the splicing-inhibited mRNA containing intron3 which contains a termination codon (calc mw 3823) was seen at the tested PNA concentrations. Furthermore, PNA2512 induced a several fold increase of p53 protein level which was not observed with the control PNA2733. This effect is ascribed to the reduction in MDM2 protein level *via *the "negative feedback regulation" mechanism [16]. Whether simply inhibiting MDM2 synthesis is most effectively achieved via targeting of splicing as described in the present report or through direct translational inhibition as previously found [20] remains to be established by further optimization and direct comparison although the data so far indicate comparable potency.

**Figure 10:**
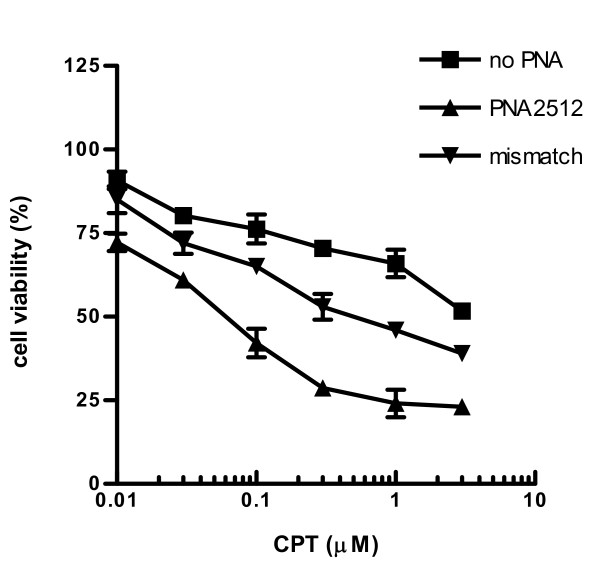
**Influence on cell viability of PNA in combination with camptothecin (CPT)**. JAR cells were transfected with PNA (PNA2512 or its mismatch (mismatch, PNA2733) at 6 μM) in the presence of LFA2000 (8 μl/ml) and CPT at the indicated concentrations for 48 h and subjected to the MTS cellular viability assay (Promega). The obtained data are normalized to the non-treated sample and shown as relative cell viability (%). In the absence of CPT cell viability was 82% and 91% in the presence of PNA2512 and mismatch PNA2733, respectively (at 6 μM). Data are mean ± S.D. of four independent experiments.

Finally, we tested the effect of PNA2512 on cell proliferation in combination with the topoisomerase I inhibitor anticancer agent camptothecin (CPT). It could be anticipated that DNA-damaging agents that activate the p53 response could act synergistically with antisense agents that activates p53 by reducing MDM2 levels, as has previously been indicated employing phosphorothioates [19] and PNA [20]. Thus the effect of PNA2512 on cell proliferation was evaluated in combination with the DNA-damaging anticancer agent CPT (Figure 10). Cells treated with CPT in combination with PNA2512 at 6 μM showed significantly lower cell viability as compared to CPT treatment alone or to CPT-treatment in combination with a mismatch PNA. We also tested lower concentrations of PNA2512 (2 and 4 μM) in combination with CPT but found no significant effect (data not shown). The results do not allow us to conclude if the effect is truly synergistic, but they do indicate that it is more than additive. However, further experiments are required for more detailed characterization of cell death induction by PNA in combination with CPT.

## Conclusions

In summary, we have identified several PNAs targeting the 5' or 3' splice sites in intron2 of mdm2 pre-mRNA which can inhibit splicing. One of the most efficient PNAs (PNA2406) is targeting the 3' splice site and is complementary to 4 bases in intron2 and 11 bases in exon3. Interestingly, PNA2512, which is targeting the 3' splice site of intron3 and is complementary to 4 bases in intron3 and 11 bases in exon4, showed both splicing inhibition as well as exon skipping. Furthermore, PNA2406 inhibited splicing of both intron2 as well as the downstream intron (intron3), whereas PNA2512 only inhibited the splicing of intron3 (and not downstream intron4). These results reflect the complexity of the splicing mechanism and thus the splicing modulation by antisense PNAs (and presumably other antisense agents as well). Further studies are required to obtain more generally applicable guidelines for designing antisense PNAs for a desired splicing modulation. Treatment of JAR cells in culture with the most potent PNA (2512) for pre-mRNA splicing modulation also affected protein expression, causing a reduction of the level of MDM2 protein and (consequently) an increase in the level of p53. Finally, this PNA increased the cytotoxicity of the anticancer camptothecin presumably caused by the PNA mediated up-regulation of p53. In conclusion these results add to the accumulating evidence that antisense mediated mRNA splice modulation have interesting prospects both within drug discovery but also as a molecular biology tool. Once more potent *in vivo *PNA delivery methods are available, the present results provide the basis for further drug discovery studies.

## Competing interests

The authors declare that they have no competing interests.

## Authors' contributions

TS and PEN designed the project; JE and TS performed the experiments; TS and PEN wrote the paper. All authors read and approved the manuscript.

## Pre-publication history

The pre-publication history for this paper can be accessed here:

http://www.biomedcentral.com/1471-2407/10/342/prepub
